# The Coming College Elections

**Published:** 1921-01-22

**Authors:** 


### THE MATRONS' AND SISTERS' DEPARTMENT.

### THE COMING COLLEGE ELECTIONS.

There are many signs that College members are going to take the elections for the Council more seriously this year than they have ever done before. They may take heart in their labours from the reflection that the question of making representative bodies really representative is the root problem which underlies democracy, and has exercised the minds of men ever since autocratic rule became distasteful to the many, without their having yet found a satisfactory solution.

The system adopted by the College for the election of the Council is that of open nomination, each member being at liberty to nominate any qualified person she pleases, provided she can obtain a promise that her nominee will serve if elected. On the surface it is a fair enough plan, but it is not working out quite as might be expected. The ordinary voter has not yet learned to vote for any but candidates she knows about. She picks out familiar names from the list, and the familiar names are usually those of matrons and doctors. London people are better known, and so Londoners are selected. Private nurses, sisters of wards, matrons of small hospitals or special institutions are seldom known to a large circle, and they do not get elected. This is the explanation of the representative defects in the College Council.

The remedy largely lies in the hands of the Centres, and must start with some concerted action about nominations. There will be twelve vacancies on the Council. How can these be filled with the greatest advantage to the College? The retiring members consist of six matrons, four doctors, one layman, and one superintendent of district nursing. Each of these categories requires to be considered separately.

An immense volume of work has been most self-denyingly and efficiently performed by the matrons on the Council. Considering their knowledge of the things which relate to nursing interests, and the fact that they have each and all passed through many successive grades of the Nursing Service on their way to their present posts, it cannot be considered that there is a preponderance of matrons on the Council. They now number twelve in all, including Scotland and Ireland. But neither the small general nor the special institutions have any, and the large and important branch of institutions under the Poor Law has but one representative on the Council. The remedy is to take steps to nominate at least four representatives of each class of these institutions, small general, special, and Poor Law. Members of the Centres should have it pointed out to them that it is their duty to cast a vote for one at least of the ladies representing each of these interests, instead of merely considering whether or no they are acquainted with the candidate. The candidates nominated under these categories ought not, of course, to be all Londoners. The different Centres have it in their power to determine that representatives shall be nominated from different parts of the country, and that when nominated they should be voted for. An admirable suggestion from one well acquainted with the difficulties of Council attendance from a distance is that each Centre should undertake to guarantee the out-of-pocket expenses of elected representatives, which often come to far more than the travelling expenses defrayed by the College itself. Meetings may necessitate a night in town, and sometimes the Councillor might have to provide a substitute for the time she is away. If such expenses were guaranteed nominations to the Council might be possible in cases where they are now declined with thanks.

An alteration in the voting-list of candidates sent to members is urgently called for. Could not the name of the Centre which backs certain candidates be printed after the candidate's name, as also the branch of the profession she represents? The The addition of the words, "Wales—Poor Law," for instance, would ensure that most Welsh and many Poor-law members would cast their votes for the candidate.

There are four medical men retiring, and the total of ten now serving is too many for a Council numbering only thirty-six. Without in the least undervaluing the aid which members of the medical profession have afforded the College, we yet do not believe it to be the desire of the members to exclude nurses from the Council by the haphazard nomination of well-known doctors whose reputation suffices to bring them election.

Of superintendents of district nurses four are too many. It would be wiser to nominate sisters or private nurses well acquainted with the circumstances which govern employment, and accept such nominees as representative of the Centres. There is ample scope for the distribution of candidates among the varied currents of nursing interests and localities, but in order to secure a good balance it is essential to take a great deal of trouble with the nominations, and issue rather more guidance for individual voters than has hitherto been afforded. It is important too to keep the voting list as short as possible. The presentation of a long list of names, probably mostly unknown to the College elector, tends to bewilderment which may end in despair.

